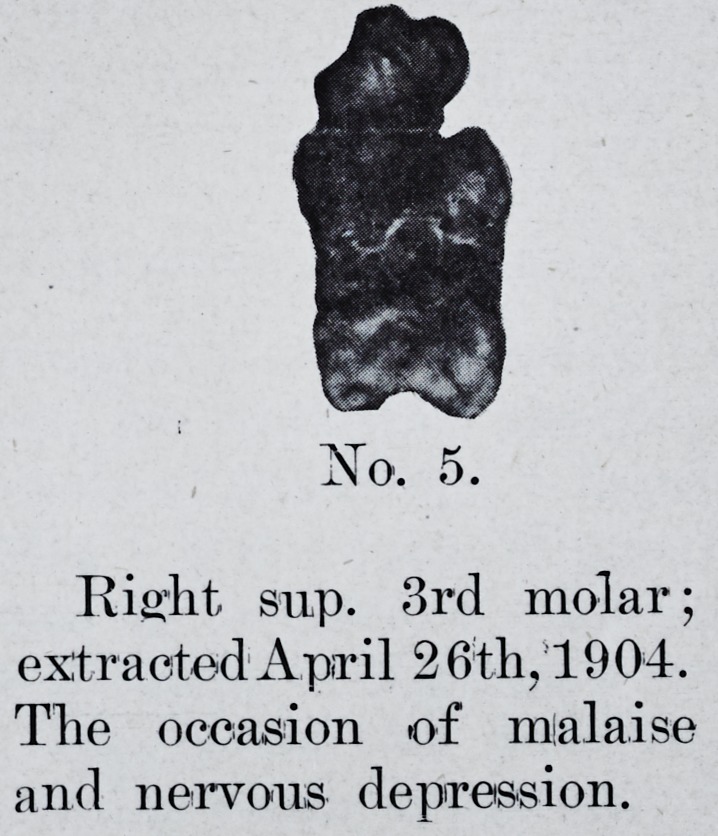# Pericemental Abscess

**Published:** 1904-12

**Authors:** D. D. Smith

**Affiliations:** Philadelphia, Pa.


### PERICEMENTAL ABSCESS.

By D. D. Smith, D. D. S., M. D.
Philadelphia, Pa.

*Read before the Philadelphia County Medical Society, Sept.* 23, 1904.

The affection here denominated pericemental abscess was first noticed by the author about 1890, and first described by him in a paper read at the Tri-union Meeting of the Virginia State, Maryland State and District of Columbia Dental Societies, held Old Point Comfort, Va., May, 1897. Prior to this all abscesses associated with the teeth and alveolus were styled alveolar abscess; the only exception to this being the occasional use of the meaningless term "blind abscess."

It was not until quite a number of these peculiar tumoric growths found on the roots of teeth—especially between the roots of molars—had been observed and noted that their distinguishing characteristics were made a subject of special interrogation and study.

### ATTENTION FIRST ARRESTED.

While treating the putrescent root-canals of a superior left bicuspid in the year 1890, two hitherto unobserved diagnostic conditions came prominently into notice. It was evident that the tooth was associated with an abscess, yet the most persistent efforts to relieve it by treatment through the root, were wholly without results; at the same time the previously cleansed and disinfected canals of the tooth remained entirely devoid of infection. Repeated dressings and medication during a period of three weeks failed to develop any odor or to make any impression whatever for the better. At the end of four weeks the tooth was extracted, when the cause of the trouble and the negative results in treatment, became apparent. Lodged in an irregular depression of the root, formed by a slight sharp bend, about one-quarter of an inch from the apex, was a glandular, fibrous tumor of the size of an ordinary pea, covered with globules of pus. The base of this tumor, or abscess, was relatively large and strongly attached to the pericemental membrane. It was entirely independent of the pulp and canals, having no communication with them whatever either at the basal attachment or at the apex. I remember well, that in reciting this feature of the case at the meeting referred to, several who took part in the discussion refused to believe that such conditions could exist.

### THE DAWNING OF RECOGNITION.

In the dawning of my observation of these pathological conditions, the recognition of them was so confused and imperfect—for in the beginning only chronic cases were apprehended—that it required several years to differentiate them from the ordinary alveolar abscess.

### THE REVELATIONS OF AN ACUTE CASE.

The observation and conduct of an *acute* case in the Spring of 1895, developed the missing diagnostic links, opened the way to a more perfect understanding of the etiology of the trouble, and suggested the nosology we have adopted.

This case is of peculiar interest, and is here cited in the hope that it may serve to fix the special pathognomonic features of the disorder. It was in connection with a second left upper molar, a tooth of typical formation, in the mouth of a young M. D.—a mouth marked, because of virulent infection on all surfaces of the teeth.

### BEGINNING OF AN ACUTE CASE.

The patient presented rather hesitatingly, complaining of an uneasy sensation only, which he hardly classed as pain, but which he located without hesitation in the offending tooth. Examination disclosed no diagnostic symptoms except a very slight loosening of the tooth and a barely distinguishable response to tapping and pressure. There being no evidence of periosteal irritation, and the pulp being alive, I was disposed to regard the complaint as having origin more in the imagination than in a real pathological state. Following an application of tincture of iodine to the gum over the affected tooth, the case was dismissed with the suggestion that the pain was probably due to some unusual or misdirected pressure in biting.

The next day the patient returned, with the local symptoms equally obscure; there was little evidence of inflammation, no swelling, no decided pain on pressure. The most prominent feature of the case at this stage was the feeling of apprehension, the decided conviction of the patient that there was something wrong with that particular tooth.

The case was a regular daily visitant for about eight days. During the first five days no strongly marked diagnostic features developed, but it excited interest, and not a little anxiety. The treatment consisted of external applications only, the quieting effect of which seemed to be most evanescent. Neither heat nor cold applied directly to the tooth and gums gave rise to any unusual sensations of pain or relief. There was no appreciable swelling, no marked periostitis or other evidence of the inflammation save the pain; this had become continuous, and was definitely located *in the tooth*.

On the fifth day a diagnosis of acute pericemental abscess was made. The tooth had become more loose and perhaps more painful; it responded more acutely to tapping and pressure, but the pulp was deemed to be alive. To asure myself that it was not implicated in the trouble, I decided upon devitalization.

(IN PARENTHESIS.)

And here parenthetically I desire to put on record a protest against much that has been written in disparagement of arsenic for the devitalization of pulps, and present the one proper method for using this valuable agent.

ARSENIC AS A DEVITALIZER.

Although wholly contrary to accepted teachings and practice, the *arsenical paste* —white arsenic, morphia acetas aa, creosote Q. S.— should *never* be placed in contact with pulp tissue; this application should always be made to intervening vital dentin.

The cavity in the tooth in which the application is to be made should be so shaped and arranged that the arsenic shall be securely confined in contact with vital, sensitive dentin only. There should be no possibility of its escape to other mouth tissues, either through the confining filling or around the margins of the cavity. If the cavity of decay cannot be made to securely confine the arsenic—and very frequently it cannot—or if it is desired to destroy the pulp in a sound tooth, a special cavity or large drill-pit should always be made *in another part* of the tooth, one in which the arsenic can be perfectly confined. The application may be made in a cavity remote from the pulp with perfect assurance, provided it is secured there in contact with sensitive dentin: (temporary gutta-percha stopping is the best confining medium).

WHY PAIN AND SYSTEMIC SYMPTOMS RESULT FROM THE USE OF ARSENIC AS A DEVITALIZER.

It is placing the arsenic upon living pulp tissue that induces the pain, and it is the escaping of the arsenic from the cavity to the pericementum and gums, through or around the confining filling, that has been and is, the cause of all supervening troubles—gum inflammations, alveolar absorption and necrosis—which result from the use of this agent.

Manipulation in accordance with the method suggested will insure painless and perfect devitalization in any tooth in from twenty-four to seventy-two hours, and that with no possible systemic injury, neither inflammatory manifestations in any contiguous tissues.

THE NARRATIVE RESUMED.

Drilling through a small crown filling into the dentin, the tooth-bone was found in an exalted state of sensibility. Assured of my diagnosis, I determined, if possible to reach the seat of the abscess and evacuate the pus in the hope of effecting a cure. An arsenical application was accordingly made to the dentin, which so perfectly destroyed the pulp that its removal was effected within thirty-six hours. This operation produced no disturbance, neither was there any incident following it. The removal of the pulp and dressing of the roots had no appreciable influence on the abscess symptoms; the soreness of the tooth, the continuous (not throbbing) pain, and the mental disturbance—a feeling of apprehension—continued unabated. I next drilled entirely through the crown, reaching the alveolus at the point which extends down between the roots. This operation was very disappointing in that it failed to evacuate any pus or to develop any odor. The third day after the opening was made upon the alveolus, there appeared unmistakable signs of infection in the left antrum. Unwilling to risk further treatment, the offending molar was extracted, when the cause of all this trouble became apparent. Just above the opening which had been made with the drill, and closely adherent to the inner side of the distal buccal root, was the peculiar but well-developed lobular sac of the abscess, with small globules of pus disseminated over its surface. The night following the removal of the tooth there was a copious discharge from the antrum through the left nostril. An opening was at once made through the alveolus into the antrum and treatment instituted through it. The antral cavity was syringed twice daily with phenol sodique, diluted about one-half with water, and at the end of two weeks the case was discharged entirely well.

WHY THIS CASE POSSESSES GENERAL INTEREST.

This acute case should possess general interest for the profession, not alone because it presents the distinguishing peculiarities and diagnostic symptoms of this affection, but more because it discloses the serious nature of the malady and points to some of its usual but unrecognized complications.

A COMMON AND SERIOUS MALADY.

It is manifest that the pathological condition we are discussing is not only far more common than has hitherto been suspected, but that the infection resulting from it is the occasion of many grave systemic diseases as yet unsuspected. It is a condition which may be readily diagnosed when in a state of activity, but when chronic and apparently inert, it may be difficult of recognition. In the guise of innocent inactivity it gives rise to perpetual infection, and frequently arouses most serious systemic maladies, conditions far more to be dreaded than its most aggravated local expressions.

MANY CASES TREATED.

During the ten years which have followed the treatment of the case cited I have seen and treated a goodly number of these abscesses, both acute and chronic, but never with encouragement or pronounced success. One of the former occurred in my own mouth in connection with a maltreated, split molar, which eventuated in an obstinate double antral infection. Another—chronic—illustrated in this article, No. 3, was the occasion of a grave head trouble—a burning pain under the scalp, attended with exhaustion and despondency. These conditions were immediately relieved through extracting the tooth. No. 4 was the cause of the development of a severe double antrum infection. No. 5 was the occasion of a marked condition of malaise, attended with mental depression and hallucinations. In the two latter cases, extraction of the offending teeth afforded immediate and pronounced relief; the antrum trouble still under treatment is progressing favorably and the malaise and mental depression eventuated in complete systemic restoration.

In but two instances, where pericemental abscess has been surely diagnosed, has there been any prolonged effort to retain and preserve the tooth. In these cases—both teeth superior molars—the abscesses have extensive basal attachment, which supplants and occupies the territory on the inner surface of the roots at their junction with the crown. These abscesses are quiescent, and have no perceptible pus discharge; while the teeth are somewhat loose, they are in use in mastication, and occasion no complaint. In both instances the teeth were experimentally retained: there is absolutely no hope of bettering their condition by treatment, either through surgical removal or lymphatic absorption.

CONFIRMATORY TESTIMONY FROM DR. FORMAD.

The histological researches of the late Dr. Formad, of the Medical Department of the University of Pennsylvania, as revealed in a private letter under date January 20th, 1898, fully confirm the status ascribed this pathological condition as a new discovery; they also stamp its nosology as correct, and reveal why it may, and does, occur indiscriminately on teeth with and without vital pulps. Caused as it is by an irritant on the outer surface of the pericemental membrane, it is wholly unaffected by any known method of treatment, either through the root, by external applications, or by surgical interference.

TEETH SPECIALLY LIABLE TO PERICEMENTAL ABSCESS.

Any tooth having a pericemental membrane, and a conformation to accumulate infection, may be the subject of pericemental abscess. Molars with large, bell-shaped crowns and constricted necks, because of their shape and location in the mouth, are more especially liable to it.

WHAT IT RESULTS FROM.

The conditions preceding its development are neither systemic inclination through "aberrant nutrition," nor a circulation floating a so-called gouty poison. It is not a result of infection from putrescent pulp tissue, nor from the prolific gaseous emanations resulting from nitrogenous decomposition within the tooth. Infection from such sources finds expression on the *inner* surface of the pericementum about the apical foramen, and eventuates in the ordinary alveolar abscess. The nucleus of this inflammation, and the resultant tumoric abscess, is in touch with some stagnant, septic irritant upon the tooth, the external surface of the pericemental membrane alone being involved.

PUS DISCHARGE SMALL.

The amount of pus disengaged from these abscesses is relatively small. It is not confined within a sac or limiting membrane, as is generally the case with an ordinary alveolar abscess, but it is sparingly distributed over the irregular surface and within the folds of the tumor-like body which forms the abscess.

HOW A PERICEMENTAL ABSCESS DISCHARGES.

The pus from a pericemental abscess never forces an outlet through the alveolus and gums in the form of a fistulous opening, neither does it come into the pulp canals through the apical foramen. It escapes at the edge of the alveolus, at the free margins of the gums—commonly between root bifurcations—or it may be found oozing around one particular root of a molar; frequently it is the palatine root of a superior molar or the distal root of a lower molar. Turgidity of the gum tissue at the point of egress marks the exact location of the abscess. No pus whatever can be obtained from a pericemental abscess in its earlier or acute stage. Even in chronic states the discharge is seldom profuse. In a goodly number of cases where the abscess had assumed a hopelessly chronic form, I have found pus in large quantities and very offensive, in a few instances in connection with lower bicuspids, but more frequently around lower molars. The incessant inflammatory action induced by this abscess not only increases its own substance, but it loosens the tooth, and through necrotic absorption forms, enlarges and deepens cavities and pits in the alveolus which become cesspools of pus prolific in infection.

The nerve centers, from my observation, are the special points attacked by the pyemic toxins from this abscess. Depression of spirits, loss of appetite, enfeebled digestion, malaise, headache, tonsular and pharyngeal inflammations, are the direct outcome. They also become the exciting cause of many far more serious troubles, both acute and chronic; among these we have found neurasthenic, gastric, gastro-intestinal, rheumatic and renal complications, including diabetes and albuminuria. From all these maladies we have not only afforded relief but effected cures by relieving septic mouth conditions, complicated with pericemental abscess.

POTENCY OF MOUTH CONDITIONS ORIGINATING SYSTEMIC INFECTION JUST IN THE DAWNING OF RECOGNITION, BY MEDICINE AND DENTISTRY.

Since the publication of the article on "*Systemic Infection Due to Natural Teeth*

*Conditions,"* in January, 1903, there have appeared two papers of marked significance by medical men: one by Dr. Robert T. Morris, of New York City, entitled, *"Infections of the Lymph-glands of the Mouth and Throat,"* and the other, *"Buccal Antisepsis,"* by Dr. E. Dunogier, Bordeaux, France. The former makes this significant and commendable remark: "We are finding at my clinic very many more infections proceeding from the teeth than are found in some other clinics," and then goes on to say, "One class of infections, very dangerous ones, have been frequently overlooked by dentists; these are infections following the removal of abscessed teeth. Patients die and the cases are not reported; they come in to be treated for pneumonia. There are patients dying this minute in this city from the result of having abscessed teeth extracted while in course of acute infection."

Dr. Dunogier speaks of "dangerous systemic results accompanying purulent conditions about the oral cavity," and recites the case of a "young man aged twenty-one, a sufferer from albuminuria for over six years," who was cured through, what seems to me, very indifferent attention to the teeth and mouth conditions, which had previously been wholly neglected. To prove positively "whether the mouth-infection had been directly concerned in the causation of albuminuria, the patient was directed to abandon temporarily all dental care. This was followed five days afterward by the reappearance of the albumin, and thus the original diagnosis was definitely established." Another case of a diabetic, is noted by Dr. Dunogier with similar treatment and like favorable results.

When the day shall dawn when medicine and dentistry shall fully recognize the human mouth as the field of most prolific and dangerous infection—the human mouth—and truly differentiate its pathologic conditons, the prevention of disease, the relief of suffering and the lengthening of the average of human life, will be accelerated a hundred fold.

If it is true, as Dr. Morris says, that in dentistry and surgery "we find whatever we are looking for," how important that we keep our eyes fixed on actual conditions. Actual conditions do not reveal that the extraction of a tooth with an ordinary alveolar abscess was ever the precursor of pneumonia. Pericemental abscess, with its pyogenic infection and enfeebling results, has undoubtedly been a predisposing factor in many cases of pneumonia. How important that medicine and dentistry distinguish between the two conditions!

Mouth conditions would appear very different to both professions if they could be viewed in the light in which they really exist. We do not need the hypothetical conditions of "thrombi" and "embolic infection," said to be induced by equally hypothetical "crushing of cancellous bone structure in tooth extraction," for the induction of systemic infection. Infection is found as a natural exudate along the whole linear gum margin about the teeth; it is found upon the twenty to thirty square inches of tooth surface in every mouth, between and under the teeth, and in crypts and pockets about roots. It is found in nasal passages, upon the tongue, on tonsils and pharynx. There is infection in the breath, in salivary and mucus sediment, in decaying food remains and in mouth debris of every kind. Add to these *normal* infectious states, the many serious pathological mouth conditions—decay in the teeth, putrescent pulp tissues, the retrograde metamorphosis of devitalized teeth, alveolar abscess, alveolar pyorrhea, and more than all the infection we are considering, pericemental abscess,—and we have a combination of infection in the human mouth which should startle the community, and rouse to highest intensity the interest of medicine and dentistry alike.

The question may arise, is pericemental abscess a state of pyorrhea? My answer is emphatically in the negative. Pericemental abscess and alveolar pyorrhea are often associated in the same mouth, and they may have similar origin, but they are separate and distinct affactions. Pericemental abscess develops from a point of irritation on the external surface of the pericementum, usually some inaccessible depression in which the infection lodges and is confined. It is generally found between the roots of double or multi-rooted teeth, or it may be found at the end of a tooth having fused roots. Alveolar pyorrhea is more commonly found in connectio with single rooted teeth; when associated with molars, the infection is between, rarely, if ever, under them.

Pericemental abscess is a characteristic tumoric growth, generally between the roots of teeth; alveolar pyorrhea is an inflammation beginning in the pericementum, at the free margin of the gums; in its progress it destroys cemental and alveolar tissue, uncovers portions of the roots of teeth, develops pus, and imparts a characteristic odor to the breath. This inflammation leaves in its train not degenerate, sero-fibrous *growths*, but broken-down tissue remains, calcic sedimentary matter, and other debris which destroys permanently all vital relations between the uncovered portions of the affected roots and the alveolus.

Pericemental abscess, virulently infectious, is incurable, except through loss of the tooth.

Alveolar pyorrhea, often equally infectious, is before the necrotic wasting of the alveolus wholly amenable to rational surgical treatment.

### DIAGNOSTIC SYMPTOMS.

The diagnostic symptoms of pericemental abscess are distinct and plain when once distinguished. In acute cases the first manifestation is pain; not severe, but continuous, and located by the patient in the offending tooth. Nervous apprehension on the part of the patient is, I believe, always incident to it. The trouble is liable to be confounded with periodontitis, but is readily distinguished from it. In acute pericemental abscess, there are no marked inflammatory symptoms in the alveolar and gum tissue; no swelling, no acute pain in response to tapping or pressure, no marked distinction between hot and cold applications, and no relief afforded by any local medication. The loosening of the tooth, slight in the beginning, becomes more and more marked as the case progresses. It will thus be noted that while this affection has been, and is, liable to be confounded with periodontitis, all the diagnostic symptoms (except the pain and the response to pressure) in the former, are exactly the opposite to those in the latter.

Chronic pericemental abscess is distinguished by absence of pain and all other active inflammatory symptoms. Occasionally, there is some hypertrophy of the gum tissue, but no marked swelling, neither soreness of the tooth on pressure. The tooth itself can generally be used in mastication without special discomfort. The escaping pus is discharged at the edge of the alveolus—between the aveolus and the gum. The point of egress may be distinguished by a peculiar lip, or mouthlike opening, marked by a circumscribed turgescent condition of the gum tissue; a condition wholly unlike the fistulous opening of a alveolar abscess.

## Figures and Tables

**Figure f1:**
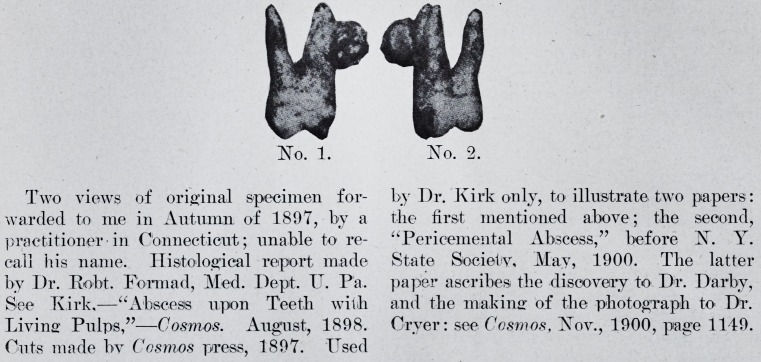


**No. 3. f2:**
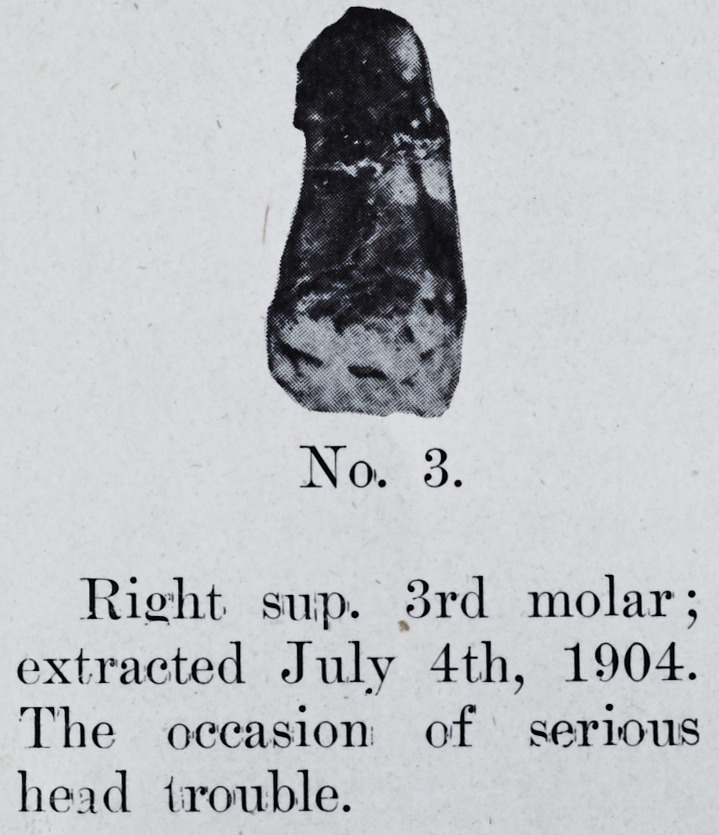


**No. 4. f3:**
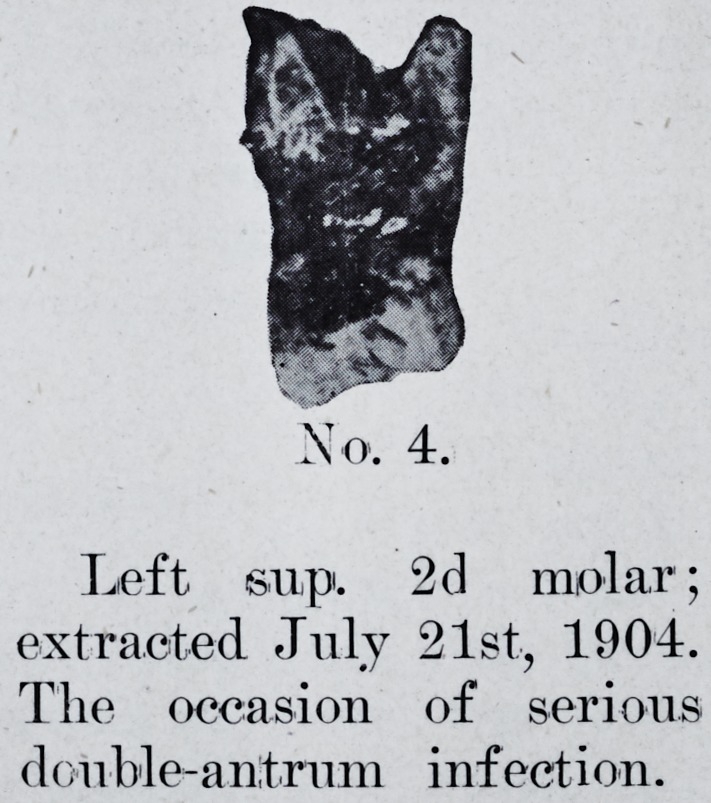


**No. 5. f4:**